# Use of Molecular Diagnostic Tools for the Identification of Species Responsible for Snakebite in Nepal: A Pilot Study

**DOI:** 10.1371/journal.pntd.0004620

**Published:** 2016-04-22

**Authors:** Sanjib Kumar Sharma, Ulrich Kuch, Patrick Höde, Laura Bruhse, Deb P. Pandey, Anup Ghimire, François Chappuis, Emilie Alirol

**Affiliations:** 1 B.P. Koirala Institute of Health Sciences, Dharan, Nepal; 2 Institute of Occupational Medicine, Social Medicine and Environmental Medicine, Goethe University, Frankfurt am Main, Germany; 3 Senckenberg Biodiversity and Climate Research Centre (BiK-F), Frankfurt am Main, Germany; 4 Senckenberg Forschungsinstitut, Frankfurt am Main, Germany; 5 Division of Tropical and Humanitarian Medicine, University Hospitals of Geneva, Geneva, Switzerland; 6 Médecins Sans Frontières UK, London, United Kingdom; University of Kelaniya, SRI LANKA

## Abstract

Snakebite is an important medical emergency in rural Nepal. Correct identification of the biting species is crucial for clinicians to choose appropriate treatment and anticipate complications. This is particularly important for neurotoxic envenoming which, depending on the snake species involved, may not respond to available antivenoms. Adequate species identification tools are lacking. This study used a combination of morphological and molecular approaches (PCR-aided DNA sequencing from swabs of bite sites) to determine the contribution of venomous and non-venomous species to the snakebite burden in southern Nepal. Out of 749 patients admitted with a history of snakebite to one of three study centres, the biting species could be identified in 194 (25.9%). Out of these, 87 had been bitten by a venomous snake, most commonly the Indian spectacled cobra (*Naja naja;* n = 42) and the common krait (*Bungarus caeruleus*; n = 22). When both morphological identification and PCR/sequencing results were available, a 100% agreement was noted. The probability of a positive PCR result was significantly lower among patients who had used inadequate “first aid” measures (e.g. tourniquets or local application of remedies). This study is the first to report the use of forensic genetics methods for snake species identification in a prospective clinical study. If high diagnostic accuracy is confirmed in larger cohorts, this method will be a very useful reference diagnostic tool for epidemiological investigations and clinical studies.

## Author Summary

Snakebite is an important medical problem in sub-tropical and tropical regions, including Nepal where tens of thousands of people are bitten every year. Snakebite can result in life-threatening envenoming, and correct identification of the biting species is crucial for care providers to choose appropriate treatment and anticipate complications. This paper explores a number of methods, including molecular techniques, to assist care providers in identifying the species responsible for bites in rural Nepal. Out of 749 patients with a history of snakebite, the biting species could be identified in 194 (25.9%). Out of these, 87 had been bitten by a venomous snake, most commonly cobras (n = 42) and kraits (n = 22). This study is the first to report the use of molecular techniques for snake species identification. The diagnostic accuracy of this method appears high but needs to be confirmed in larger studies.

## Introduction

In rural Nepal snakebite is an important public health problem. A survey conducted in the 1980s showed that about 20’000 people were bitten each year, resulting in over 1’000 deaths [1]. These official figures, however, significantly underestimate the true burden [2–7]. Annual incidence and mortality figures of 1’162/100’000 and 162/100’000, respectively, have been reported in some regions of Nepal [4]. Children are among the primary victims [2,8,9]. Most snakebites occur in the southern plains of Terai, a region with a hot tropical climate and high population density. Most bites occur during the rainy season, from June to September, which corresponds to the peak period for agricultural work.

Eighty nine snake species have been recorded in Nepal, of which 17 are known to be venomous [10]. The Elapidae family includes two species of cobra (*Naja naja* and *Naja kaouthia*), the king cobra (*Ophiophagus hannah*), one species of coral snake (*Sinomicrurus macclellandii*) and six species of krait (*Bungarus bungaroides*, *Bungarus caeruleus*, *Bungarus fasciatus*, *Bungarus lividus*, *Bungarus niger*, and *Bungarus walli*). The Viperidae are represented by seven species. The most dangerous of these, Russell’s viper (*Daboia russelii*), appears to be rare in Nepal. Pitvipers (*Gloydius himalayanus*, *Himalayophis tibetanus*, *Ovophis monticola*, *Protobothrops* spp. *Trimeresurus albolabris* and *Trimeresurus septentrionalis*), on the other hand, are widespread from the lowlands to the high mountains. Non-venomous species are also very common and may be involved in bites. Some of these non-venomous species are easily mistaken for venomous ones [10]. For example, rat snakes (*Ptyas* and *Coelognathus* species) may be confused with cobras, while wolf snakes, which are common inside and around houses, have a colour pattern similar to that of kraits [11–13]. Bites can therefore be inflicted by a variety of species, in all kinds of environments. Neither the geographical distribution of these species nor their contribution to snakebite mortality and morbidity have been systematically studied in Nepal.

Snakebite envenoming can be life-threatening, and recognizing early signs of systemic envenoming is crucial for the optimal management of patients. Depending on the species of snakes, different organs and tissues can be affected [14]. In envenoming following the bites of elapid snakes, neurotoxicity with progressive descending paralysis is characteristic, and patients usually die of respiratory failure if not adequately ventilated [15–17]. Clinical prognosis and response to antivenom depend on the species, hence knowing which snake is responsible for the bite is of primary importance [14]. In South Asia appropriate tools for species identification are not available. The snake is rarely seen, and if it is, its description by the victim is often misleading [18]. Hospital personnel are generally not trained to properly identify the biting species, even when it is killed and brought by the victim [19–21]. The morphological resemblance of venomous by non-venomous species complicates this task.

Several approaches have been investigated to improve snake species identification in peripheral health centres. Immunodiagnosis of circulating venom antigen can be used to identify the biting species or ‘immunogroups’ of antigenically similar, cross-reacting venoms [22–25], but commercial point-of-care venom detection kits have been marketed only in Australia, for species occurring on that continent [26]. In Sri Lanka, syndromic approaches have been proposed [20] and clinical scores developed based on a systematic analysis of features of envenom envenoming in cases where snake specimens brought to hospital were expertly identified [27]. However, the extent to which these approaches can also be applied to the Nepal setting is not known. The present study aimed at investigating the accuracy, feasibility and usefulness of different, complementary approaches to species identification in patients bitten by snakes in rural Nepal. The main objectives were (1) to clarify the contribution of different snake species causing envenoming and non-envenoming bites through morphological analysis of preserved snake specimens and the use of forensic methods for DNA-based identification, and (2) to explore the utility, diagnostic performance and feasibility of DNA-based identification by forensic methods.

## Methods

### Study setting and population

The study was conducted in three centres, namely the Snake Bite Treatment Centre of Damak Red Cross Society and the Snake Bite Management Centre of Charali, both in Jhapa district, and Bharatpur District Hospital, Bharatpur, Chitwan district. All three centres are located in the Terai lowland region of Nepal.

All snakebite victims presenting to the Damak Red Cross Centre and to the Charali Snake Bite Management Centre during the study period were offered to participate in the study, irrespective of the presence and nature of envenoming signs. Hence, victims of bites by elapids, viperids and non-venomous snakes were included. Patients were excluded from the study if they were below 5 years of age or if they had already received antivenom prior to admission. Patients unable or unwilling to give consent were also excluded. In Bharatpur District Hospital, additional exclusion criteria were applied due to the contemporary conduct of a randomized controlled trial on neurotoxic envenoming (clinicaltrials.gov number NCT01284855). In this centre, only snakebite patients presenting with signs of systemic neurotoxic envenoming were included in the study. Those presenting more than 24 hours after the bite, pregnant or breastfeeding women and patients with proven viper bite envenoming were excluded. The recruitment period in Bharatpur was shorter and extended from 1^st^ April 2011 to 31^st^ October 2012.

### Species identification

Dead snakes brought by bite victims were systematically labelled with patient number, initials, date of birth and date of admission, and preserved in 70% ethanol. Morphological identification was conducted by taxonomic experts (UK, DP) who remained blind to the circumstances of bites and to envenoming status of the victims. For each preserved specimen, morphological characters of scalation and dentition were analysed by comparison to relevant reference specimens in museum collections and an existing database [28,29]. Additionally, genetic information from tissue samples of killed snakes were obtained in the form of DNA ‘barcodes’ of the mitochondrial cytochrome *b* gene [28,29].

### Molecular analysis

Whenever the bite site could be located, trace DNA of the biting snake was collected by rubbing the cotton swab of a Prionix evidence collection tube on the bite site (see Standard Operating Procedure in supplementary material). The sample was left to dry at room temperature. For DNA extraction, one half of the cotton bud was cut off and subjected to lysis in CTAB (cetyl trimethylammonium bromide) buffer with proteinase K at 56°C, followed by one extraction with phenol-chloroform-isoamyl alcohol and then with chloroform-isoamyl alcohol. DNA was precipitated with 98% ethanol and 3M sodium acetate (pH 5,2) and stored overnight at -20°C before washing with 70% ethanol, drying and dissolving in TE-buffer. For PCR we used primers flanking a 400 bp sequence of the mitochondrial cytochrome *b* gene (*cytb*) which preferentially amplify snake rather than human *cytb* sequences. For nested PCR we designed primers with *cytb* binding sites upstream and downstream of the PCR primers. PCR was performed in 30 μl volumes containing 6 μl of template DNA solution, 0.3 pM of each primer, 83 pM of each dNTP, 3 μl 10 × PCR buffer without MgCl_2_ (Fermentas), 3.25 mM MgCl_2_, 1 unit TrueStart Hot Start *Taq* polymerase (Fermentas), and 13.7 μl H_2_O. PCR products were visualized by SYBRGreen (Invitrogen) staining in 1% agarose gels and ultraviolet (302 nm) light illumination. Their lengths were estimated using a 100 bp ladder (Fermentas). Cycle sequencing of both strands was performed with 0.16 μl of BigDyeTerminator 3.1 reaction mix (Applied Biosystems), 1 μl primer, 1.92 μl 5 × sequencing buffer (Applied Biosystems) and 5.92 μl milliQ H_2_O (Millipore). Products of the sequencing reaction were separated on an ABI 3730 sequencer (Applied Biosystems) operated with a 50 cm capillary, 8 sec injection time and 1500 V injection voltage. After visual checks of electropherograms, correction of base-calls and comparison of complementary strands using BioEdit and SequenceScanner software, sequences were submitted to BLAST searches for comparison with the sequences in the GenBank, ENA and DDBJ nucleotide sequence database to determine the snake species. New DNA sequences obtained in the course of this study were deposited in this database. A full description of the methods is included in Melaun and Kuch [30].

### Features of envenoming

Local envenoming was defined as the presence of one or more of the following: (1) necrosis, (2) bullae or blisters, (3) enlarged regional lymph nodes plus either local bleeding or ecchymosis or swelling and (4) swelling extending at least halfway between two articulations. Snakebite victims presenting with moderate local swelling (i.e., not extending farther than one articulation) were not considered as locally envenomed. Systemic envenoming was defined as the presence of one or more of the following: (1) Incoagulable blood as indicated by the 20 Minutes Whole Blood Clotting Test (20’WBCT), (2) spontaneous and continuous bleeding from the bite site, IV line, gums or old wound, (3) gastrointestinal bleeding or blood in urine, (4) neurotoxic sign(s) including inability to frown, bilateral ptosis, inability to open the mouth, inability to protrude the tongue beyond incisors, inability to clear secretions, broken neck sign, skeletal muscle weakness, gag reflex loss and paradoxical breathing. Neurotoxic envenoming was defined as the presence of one or more of the above-mentioned neurotoxic signs. Envenoming status was determined retrospectively, by one of the authors (EA) who was blinded to the results of snake species identification.

### Clinical management of patients

The clinical management of snakebite victims followed the Nepal national protocol and WHO SEARO guidelines [14]. All patients presenting with systemic envenoming received antivenom, as did patients presenting with severe local envenoming (e.g., edema extending over full limb). Antivenoms available in Nepal are all manufactured in India and target four species, the Indian spectacled cobra (*Naja naja*), the common Indian krait (*Bungarus caeruleus*), the saw-scaled viper (*Echis carinatus*) and Russell’s viper (*Daboia russelii*). Additional treatments included anticholinesterases and assisted ventilation for those patients experiencing respiratory paralysis. Patients who were asymptomatic on admission were kept under observation for 24 hours.

### Data collection and analyses

A standard Case Report Form (CRF) was designed to prospectively collect data on the circumstances of the bite, the participants’ demographic characteristics, and the clinical features on admission. First aid measures, whether appropriate or not, were recorded, as well as features of clinical management after admission. To ensure harmonization of data collection, study staff were trained on CRF completion and were instructed to follow Standard Operating Procedures (SOP). Separate forms were used for the results of morphological identification and molecular analysis. As these analyses were performed well after recruitment was over, their results did not influence the assessment of snakebite victims by the care-providers. The researchers performing the PCR and DNA sequence analyses were also blind to the results of morphological identification and patient data.

Characteristics on admission were described using percentages for categorical variables and by calculating means and standard deviations or median and Inter Quartile Ranges (IQR) for continuous variables. The Case Fatality Rate (CFR) was calculated as the percentage of patients who died among those showing signs of envenoming. Descriptive analyses were restricted to participants with snake species identified by PCR or morphology. Determinants of PCR positivity were analysed for all patients for whom a PCR was done, as follows: continuous variables were compared using Student’s t test, or the Wilcoxon- rank sum test for non-normally distributed variables. Categorical variables were compared using the Χ^2^ test with continuity correction or the Fisher exact test, as appropriate. For categorical variables with more than one category, a test for trend was used. Associations were examined at the p<0.05 level of significance. To assess the probability of a positive PCR, Risk Ratios (RR) with 95% Confidence Interval (95% CI) were calculated.

### Ethical aspects

The study was conducted in accordance with the Declaration of Helsinki 1964, as revised in Seoul, 2008, and in compliance with the protocol, Good Clinical Practices (GCP) and Nepal regulatory requirements. The B.P. Koirala Institute of Health Sciences (BPKIHS) Ethics Committee, the Nepal National Health Research Council (NHRC) and Geneva University Hospital Ethics Committee approved the study prior to its start. All participants provided written informed consent before being included in the study.

## Results

**Fig 1:**
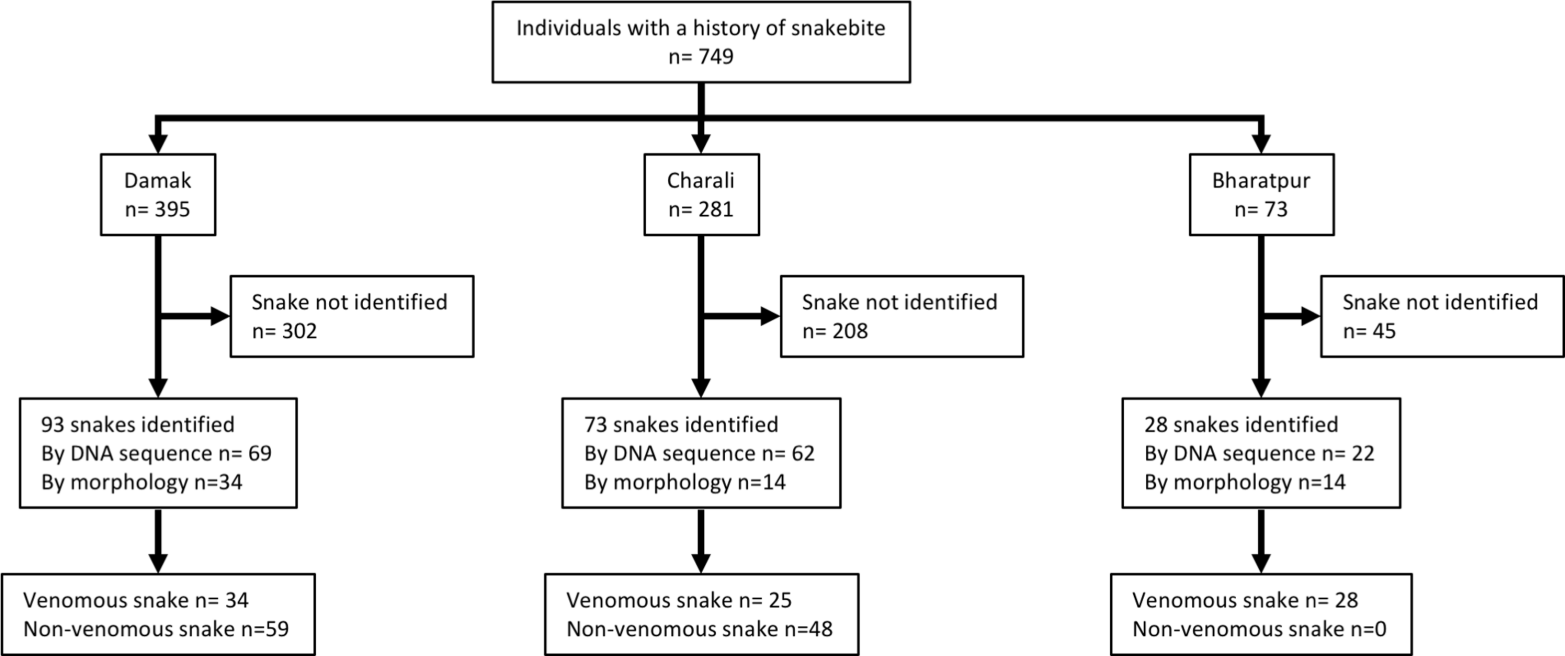
Flow diagram showing numbers of individuals screened and included in each study centre. Between 01/04/2010 and the 31/10/2012, 749 victims of snakebite were included in the study and the snake species responsible for the bite could be ascertained in 194 cases.

Between the 1^st^ of April 2010 and the 31^st^ of October 2012, a total of 749 patients were found eligible to be included in the study (Fig 1).

The responsible snake species could be ascertained for 194 (25.9%) patients. In total 722 snakebite victims presented with fang marks, and swabs were collected and analysed from 565 of them. PCR and DNA sequencing yielded a positive result in 153 out of these (27.1%). Sixty two snakes were brought by the victim and all were morphologically identified. Collectively the snake species could be determined in 87 (32.9%) of 264 envenomed patients and in 107 (22.1%) of 485 non-envenomed patients. Table 1 lists the species identified in all three centers. Among the non-venomous species, the checkered keelback (*Xenochrophis piscator*) was responsible for the majority of bites. Among the venomous species identified the most common were the spectacled cobra (*N*. *naja*) and common krait (*B*. *caeruleus*). There was a very clear difference between study sites. Most *B*. *caeruleus* specimens (20 out of 22) were brought in Bharatpur, while most *N*. *naja* (35 out of 42) came from either Charali or Damak. In 21 cases, both morphological identification and a snake DNA sequence were available. In these cases there was a 100% agreement between the two identification methods.

**Table 1:** List of species responsible for snakebite in southern Nepal between April 2010 and October 2012 (n = 194). Species were identified either by morphological examination of preserved specimen or through PCR and DNA sequencing performed using bite-site swabs.

	Damak (n = 93)			Charali (n = 73)			Bharatpur (n = 28)		
Species name (*scientific name*)	PCR	Morpho	Both	PCR	Morpho	Both	PCR	Morpho	Both
Non-venomous species n = 107							NA	NA	NA
Common wolf snake (*Lycodon aulicus*) n = 11	2	7	1	1	2	0			
Trinket snake (*Coelognathus helena*) n = 2	0	1	0	0	1	0			
Indian rat snake (*Ptyas mucosa*) n = 3	1	1	0	1	0	0			
Mock viper (*Psammodynastes pulverulentus*) n = 2	0	1	0	0	1	0			
Checkered keelback (*Xenochrophis piscator*) n = 88	45	5	4	41	2	1			
Spotted keelback water snake (*Xenochrophis punctulatus*) n = 1	1	0	0	0	0	0			
**Venomous species n = 87**									
Montain pit viper (*Ovophis monticola*) n = 5	1	0	0	4	1	1	0	0	0
Unidentified pit viper (*Trimeresurus* sp.) n = 3	0	1	0	1	1	0	0	0	0
White-lipped pit viper (*Trimeresurus albolabris*) n = 5	2	1	0	2	0	0	0	0	0
Pope’s tree viper (*Trimeresurus popeiorum*) n = 1	0	0	0	1	0	0	0	0	0
Spectacled cobra (*Naja naja*) n = 42	16	15	5	7	3	1	4	4	1
Unidentified cobra (*Naja* sp.) n = 1	0	1	0	0	0	0	0	0	0
Monocellate cobra (*Naja kaouthia*) n = 1	0	0	0	0	0	0	1	0	0
King cobra (*Ophiophagus hannah*) n = 1	1	0	0	0	0	0	0	0	0
Common krait (*Bungarus caeruleus*) n = 22	0	0	0	2	0	0	17	10	7
Greater black krait (*Bungarus niger*) n = 1	0	0	0	0	1	0	0	0	0
Lesser black krait (*Bungarus lividus*) n = 5	0	1	0	2	2	0	0	0	0

### Epidemiological characteristics

The baseline characteristics and circumstances of the bites of the 194 snakebite victims for whom a species could be ascertained, are summarized in Table 2. The majority of snakebite victims were male (52.6%) and the median age was 30.8 years (IQR = 18–40). There were 28 (14.4%) children (5–15 years). A clear seasonal pattern was observed in all three study sites, with the incidence of bites being higher during the rainy season (June to September).

**Table 2:** Circumstances of the bite and baseline characteristics of victims of snakebite in southern Nepal between April 2010 and October 2012 (n = 194).

Patient characteristics	
Sex	
Female	92 (47.4%)
Male	102 (52.6%)
Occupation	
Farmer	75 (39.9%)
Student	58 (30.9%)
Housewife	37 (19.7%)
Commercial	3 (1.6%)
Driver	4 (2.1%)
None	0
Other	11 (5.9%)
Season of bite	
Dry season (October to May)	83 (42.8%)
Rainy season (June to September)	111 (57.2%)
Time of bite	
Night (18:00 to 05:59)	111 (57.5%)
Day (06:00 to 17:59)	82 (42.5%)
Transport used to reach centre	
Motorcycle	90 (63.8%)
Ambulance	32 (22.7%)
Public transport	13 (9.2%)
Car	2 (1.4%)
Other	4 (2.8%)
Location at time of bite	
Indoors	64 (33%)
Outdoors	130 (67%)
Activity at time of bite	
Walking outdoors	76 (39.4%)
Working in the field	26 (13.5%)
Working elsewhere	28 (14.5%)
Resting indoors	29 (15%)
Collecting grass/wood	14 (7.3%)
Playing	4 (2.1%)
Feeding cattle	4 (2.1%)
Bathing or fishing	3 (1.6%)
Other	9 (4.7%)
Visited traditional healer	
Yes	4 (2.1%)
No	190 (97.9%)
Applied first aid measures	
Yes	174 (89.7%)
No	20 (10.3%)
Type of first aid measures	
Tourniquet	170 (87.6%)
Ingested chilly	6 (46.2%)
Applied herbs on bite site	1 (7.7%)
Bandage	4 (30.8%)
Incisions	0

Missing values are n = 6 (occupation), n = 1 (time of bite), n = 53 (transport), n = 1 (activity at time of bite)

The median time needed to reach the treatment centre was 85 minutes (IQR = 50 to 120 minutes), with a longer time observed in Bharatpur (194 minutes; IQR: 11 to 269 minutes). Only four (2.1%) snakebite victims visited a traditional healer before reaching the study centre. Use of first aid measures was more common in Damak (n = 91/93; 97.8%) and Charali (n = 72; 98.6%) compared to Bharatpur (n = 11; 39.5%). Tourniquets were by far the most common first aid method applied.

S1 Table compares baseline characteristics, circumstances of the bite and first aid measures between snakebite victims with identified and unidentified snake species.

### Clinical features on admission

Among snakebite victims presenting to the treatment centres in Damak or Charali during the study period (n = 676), 191 (27.4%) showed signs of envenoming. Twenty three (24.7%) patients in Damak and four (5.5%) in Charali presented with neurotoxic signs. As expected by local inclusion criteria, all 73 patients recruited in Bharatpur had signs of systemic neurotoxic envenoming on admission, and among those, seven also had local signs (see S1 Text).

The signs and symptoms presented on admission by the 194 snakebite victims for whom a species identity could be ascertained are described in Table 3. Among 87 victims for whom a venomous species was identified, 16 (18.4%) had not developed any sign of envenoming. Among the 73 patients for whom an elapid snakebite could be ascertained, 64 (87.7%) developed signs of envenoming. As expected, none of the 107 patients bitten by non-venomous species exhibited local or systemic signs of envenoming.

**Table 3:** Clinical features on admission of victims of snakebite in southern Nepal between April 2010 and October 2012 (n=194). Initial assessment of snakebite victims included vital signs, patients’ complaints and standardized evaluation of envenoming signs.

Patient characteristics	Venomous species identified (n = 87)	Non-venomous species identified (n = 107)
Envenoming		
Local signs	4 (5.6%)	
Systemic signs	38 (53.5%)	
Both	29 (40.8%)	
None	123 (63.4%)	
Level of consciousness		
Alert	84 (96.6%)	107 (100%)
Responsive to voice	2 (2.3%)	0
Responsive to pain	1 (1.1%)	0
Unresponsive	0	0
Symptoms on admission (as reported by the patient)		
Vomiting	13 (14.9%)	8 (7.5%)
Diarrhea	3 (3.4%)	1 (0.9%)
Difficulty in breathing	4 (4.6%)	0
Pain	60 (69%)	16 (15%)
At the bite site	37 (67.3%)	15 (100%)
Abdominal pain	11 (20%)	1 (6.7%)
Double vision	2 (2.3%)	0
Local signs on admission		
Fang marks	83 (95.4%)	107 (100%)
Swelling	41 (47.1%)	2 (1.9%)
Local bleeding	31 (35.6%)	29 (27.1%)
Ecchymosis	18 (20.7%)	0
Necrosis	8 (9.2%)	0
Bullae	1 (1.1%)	0
Palpable regional lymph node	3 (8.8%)	0
Haemotoxic signs		
Incoagulable blood	7 (8%)	0
IV site bleeding	0	1 (0.9%)
Neurotoxic signs and symptoms		
Inability to frown	9 (10.3%)	0
Bilateral ptosis	54 (62.1%)	0
Inability to open mouth	3 (3.4%)	0
Inability to protrude tongue	5 (5.7%)	0
Inability to swallow	26 (29.9%)	0
Muscle weakness	0	0
External ophthalmoplegia	5 (5.7%)	0
Pupil not reacting to light	18 (20.7%)	7 (6.5%)
Speech difficulties	0	0
Broken neck sign	0	0
Loss of gag reflex	0	0

Missing values are n = 1 (external ophthalmoplegia), n = 53 (palpable regional lymph nodes), n = 124 (pain location)

Swelling of the bitten limb was present in 43 out of 194 patients (22.2%), and bleeding from the bite site was present in 60 (30.9%). Of note, both signs also occurred in patients bitten by non-venomous species, in particular 25 out of 88 *X*. *piscator* bite cases (28.4%) presented with bleeding from the bite site.

The 20 Minutes Whole Blood Clotting test was performed on all victims presenting to one of the study centres during the study period. Out of those with incoagulable blood on admission, the snake species could be determined in 7 cases: 5 were *N*. *naja* and 2 were white-lipped pit vipers (*Trimeresurus* cf. *albolabris*). No snakebite victim presented with gum bleeding, gut bleeding, or blood in urine on admission.

S2 Table compares clinical features on admission of snakebite victims with identified and unidentified snake species.

### Factors affecting PCR positive rate

As the number of cases in which the snake species could be identified by PCR sequencing was low, we investigated baseline characteristics and circumstances of the bite that may influence the sensitivity of this method. Results are summarized in Table 4. The median time to reach the center was significantly longer in patients with a negative PCR. The probability of a positive PCR result was significantly lower among patients who had used first aid measures. In particular, applying local remedies (e.g., herbs, honey, etc.) was associated with a 2-fold decrease in the probability of a positive PCR. Patients bitten on the upper limb were 60% less likely to have a positive PCR compared to those bitten on the lower limb. Local bleeding also increased the probability of a positive PCR by 1.45 fold. Whereas bite by a venomous species did not affect the chances of a positive PCR (RR = 1.004, 95%CI: 0.893–1.128), showing signs of envenoming significantly increased the chances of a positive result (RR = 1.444, 95% CI: 1.091–1.912, p = 0.017).

**Table 4:** Factors associated with a positive PCR among 565 snake bite victims. Unadjusted Risk Ratio (RR) and their 95% Confidence Interval (95% CI) were calculated with respect to the baseline category, i.e., absence of the risk factor (RR = 1).

Patient characteristics	PCR positive (n = 153)	PCR negative (n = 412)	P value	Crude RR	95% CI
Season of bite					
Rainy season (June to September)	84 (25.5%)	245 (74.5%)		1	
Dry season (October to May)	69 (29.2%)	167 (70.8%)	0.378	1.145	0.873–1.502
Time of bite					
Day	62 (23.4%)	203 (76.6%)		1	
Night	91 (31.1%)	202 (68.9%)	0.053	1.327	1.007–1.751
Time to reach					
Median time to reach (IQR)	1:05 (0:45–1:30)	1:30 (1:00–2:30)	0.003		
Visited traditional healer					
No	149 (28.1%)	382 (71.9%)			
Yes	4 (11.8%)	30 (88.2%)	0.045	0.419	0.162–1.066
Site of bite					
Lower limb	105 (28.8%)	259 (71.2%)		1	
Upper limb	16 (11.4%)	124 (88.6%)		0.396	0.243–0.645
Other	0	2 (100%)	<0.001	-	-
First Aid					
No	15 (41.7%)	21 (58.3%)		1	
Yes	138 (26.1%)	390 (73.9%)	0.067	0.627	0.415–0.947
Used tourniquet					
No	18 (45%)	22 (55%)		1	
Yes	135 (25.8%)	389 (74.2%)	0.014	0.573	0.395–0.831
Applied local remedy on bite site					
No	111 (24.5%)	326 (74.6%)		1	
Yes	9 (13.2%)	59 (86.8%)	0.031	0.521	0.278–0.978
Washed bite site					
No	79 (28.5%)	198 (71.5%)		1	
Yes	41 (17.8%)	189 (82.2%)	0.005	0.625	0.447–0.873
Local signs on admission					
Swelling					
No	125 (28.7%)	311 (71.3%)		1	
Yes	28 (21.7%)	101 (78.3%)	0.147	0.757	0.528–1.085
Local bleeding					
No	106 (24.5%)	327 (75.5%)		1	
Yes	47 (35.6%)	85 (64.4%)	0.016	1.454	1.096–1.930
Ecchymosis					
No	141 (26.1%)	393 (73.6%)		1	
Yes	12 (40%)	18 (60%)	0.137	1.515	0.956–2.401

Missing values n = 7 (time of bite), n = 1 (first aid and tourniquet use), n = 34 (site of bite), n = 58 (washed bite), n = 60 (applied local remedies)

### Management and outcome

A total of 67 (34.5%) snakebite victims received antivenom. Eight were put under mechanical ventilation and nine were transferred to a tertiary care centre. Among patients treated with antivenom the median dose was 2 vials (inter-quartile-range = 1–3).

In total there were 5 deaths (Case Fatality Rate = 5/194 = 2.6%): one in Damak, one in Charali and three in Bharatpur). Of these 5 deaths, 4 had been bitten by a *B*. *caeruleus* and 1 by a *N*. *naja*.

## Discussion

Between the 1st of April 2010 and the 31st of October 2012, 749 individuals with a history of snakebite presented to one of three study centres in southern Nepal. In 194 (25.9%) patients, the responsible snake species could be ascertained, by either morphological identification or PCR plus DNA sequencing. Most species identified were non-venomous ones. The non-venomous checkered keelback (*X*. *piscator*) was the most frequently identified species, followed by the spectacled cobra (*N*. *naja*) and the common krait (*B*. *caeruleus*). Other venomous species contributing to the snakebite burden in this study comprised several pitvipers (*O*. *monticola*, *Trimeresurus* sp., *T*. *albolabris*, and *T*. *popeiorum*) as well as various additional elapid snakes, including the first cases of envenoming by the greater black krait (*Bungarus niger*) and the king cobra (*O*. *hannah*) ever reported in Nepal.

As few victims (11.6%) brought the dead snakes to the study centres, the use of PCR to amplify snake trace DNA from bite-site swabs increased to 25.9% the proportion of victims for whom the snake species could be ascertained. Morphological identification of preserved specimens by a qualified herpetologist is the gold standard for species identification. However, this method is seldom used, as snakes are rarely captured and preserved, and as care-providers working in snakebite treatment centres generally lack the appropriate expertise [19–21]. Alternative approaches must therefore be developed to complement morphological identification. Molecular techniques have shown promising results in animal models [30–33], and the present study shows that PCR amplification of a mitochondrial gene region from snake trace DNA is feasible in field setting. Sampling is straight forward and requires minimal training (see SOP in supplementary material), and the storage and transport conditions for the Prionix evidence collection tubes (room temperature and protected from light) can easily be met.

The PCR yielded a positive result in 27.1% of the cases only. This could probably be improved if several factors shown in this study to be associated with a lower sensitivity, such as inappropriate first-aid measures (use of tourniquet or application of local remedies on the bite site) or prolonged time to reach the treatment centre, were corrected or improved by public health interventions. Although the sensitivity of the PCR did not seem to be affected by whether the species was venomous or not, envenoming status did have an effect. This may be due to confounding effects of venom injection. Bites associated with venom injection may go deeper in tissues, and snakes may deposit more trace DNA on bite sites during envenoming bites compared to ‘dry’ bites or bites by non-venomous snakes e.g., snake DNA can also be recovered from snake venom [33].

In all cases where both snake morphological identification and DNA sequence from the bite-site swab were available (n = 21), a 100% agreement was observed between the two methods, suggesting a high specificity of molecular identification. An on-going study conducted by the authors is expected to complement this preliminary data and validate PCR-aided sequencing of snake trace DNA on a larger cohort of patients. Although molecular tools are not yet appropriate for point-of-care (POC) testing and hence cannot be used to guide clinical management, the encouraging results presented here, if confirmed in larger studies, suggest that they could be used as reference tests in future epidemiological and clinical studies. Progress in the development and validation of POC tests for snake species has indeed been hindered by the difficult implementation of the diagnostic gold standard (morphological identification) in rural regions where most bites occur. Besides, molecular tools could be very useful in clarifying the contribution of different snake species to the snakebite burden, and help identify new medically important species.

The species found to have caused neurotoxicity in this study were the two species of cobra (*N*. *naja* and *N*. *kaouthia*) and three different species of krait (*B*. *caeruleus*, *B*. *lividus*, and *B*. *niger*). Of these, only two (*N*. *naja* and *B*. *caeruleus*) are included in the production of the Indian polyvalent antivenoms that are available in Nepal. No pre-clinical data exist on the efficacy of Indian polyvalent antivenoms to neutralize the venoms of these two species in Nepal, and no clinical data have been published so far. In this study, patients bitten by a krait had significantly higher chances of being put under mechanical ventilation and being transferred to an intensive care unit compared to those being bitten by a cobra (see supplementary information). This is consistent with published literature which suggests that krait bites often result in poorer outcomes for patients, and high mortality rates [34,35]. The efficacy of the available Indian antivenom in reversing envenoming by kraits is increasingly being questioned, and several case series have reported little or no benefit of immunotherapy [17,36–38].

The frequency of neurotoxicity observed in our study is consistent with elapid snake species being most commonly involved in envenoming bites in Nepal. The fact that we did not observe late-appearing signs such as broken neck sign, muscle weakness and loss of gag reflex may be due to the early presentation of victims to the health centre and the prompt initiation of antivenom therapy. Interestingly, among those patients who presented with incoagulable blood on admission were five victims of *N*. *naja* bites. This is not the first report of apparent coagulopathy following bites by this species [39], and a few in vitro studies have reported anticoagulant activities in *N*. *naja* venoms [40–42], however, these results need to be interpreted with caution. In fact, the 20 Minutes Whole Blood Clotting Test can give erroneous results if performed incorrectly, in particular if the tubes used bear traces of detergent [14].

The present study has several limitations, the principal one being that the study population differed between study sites. Snakebite victims admitted to Bharatpur District Hospital were only included if they presented with signs of neurotoxic envenoming. It is therefore not surprising that all snakes identified in this centre were venomous, resulting in an overestimation of the contribution of venomous species (and in particular elapids) to the snakebite burden. When Bharatpur was excluded from the analysis, venomous species accounted for only 35.6% of identified bites. The checkered keelback and the spectacled cobra remained the most common species identified (see supplementary information). Another limitation relates to the geographical coverage of the study. The list of species identified here is not representative of all snakes causing bites in Nepal. The three study centres are located in the lowlands of the Terai region where most snakebites occur, but their catchment areas do not cover all of Nepal’s great biogeographical diversity. In particular, species found in mountain regions (although present, e.g., *O*. *monticola*) were probably under-represented in our study.

The fact that the biting species could be identified only in a relatively small proportion of patients (25.9%) could in theory lead to bias. Although we cannot exclude that some selection bias occurred in the present study, its impact is likely to be minimal. We compared bite circumstances and baseline characteristics of snakebite victims with or without identification of snake species (S1 and S2 Tables). Differences were seen with regard to season of bite, location and activity at the time of bite and consultation of a traditional healer. However, the magnitude of these differences was minimal. Moreover, the epidemiological characteristics of our study population are consistent with other published reports [2–5,8,43,44], further ruling out the possibility of selection bias and reaffirming the external validity of our findings.

Finally, morphological identification and molecular analysis results were both available in only 21 cases, limiting our ability to evaluate the diagnostic performance of the molecular diagnosis method. Findings presented here thus need to be interpreted with caution. A follow-up prospective validation study is ongoing in Nepal and Myanmar to address this issue.

Snakebite envenoming is an important health problem in Nepal, accounting for up to 39.7% of poisoning cases admitted to emergency units in certain regions [6]. Neurotoxicity following the bites of elapid snakes is of particular concern. This study for the first time addresses the distribution and medical importance of snake species contributing to the burden of snakebite in Nepal. It provides crucial information for clinicians and health workers involved in the management of snakebite victims in Nepal. It notably highlights that the majority of bites are caused by non-venomous snakes, and that the diversity of venomous snake species involved in bites is greater than previously believed. Finally, this study provides initial evidence on the utility of forensic DNA-based methods in the identification of biting snake species.

## Supporting Information

S1 Checklist STARD checklist. (DOC)

S1 Text no caption. (DOCX)

S2 Text Standard Operating Procedure (SOP) for the collection, storage and shipment of swab samples. (PDF)

S1 Table Comparison of baseline characteristics, circumstances of the bite and first-aid measures between snake bite victims with identified (n = 194) and unidentified (n = 555) snake species. (DOCX)

S2 Table Comparison of clinical features on admission of victims of snake bite with identified (n = 194) and unidentified (n = 555) snake species. (DOCX)
