# Supplementary material for: Use of Molecular Diagnostic Tools for the Identification of Species Responsible for Snakebite in Nepal: A Pilot Study
Source: PLoS Negl Trop Dis. 2016 Apr 22;10(4):e0004620. doi: 10.1371/journal.pntd.0004620 (PMC4841570; doi:10.1371/journal.pntd.0004620)
Supplement: S1 Text — (DOCX) [file pntd.0004620.s002.docx]

- Among snakebite victims presenting to the Damak or Charali centres during the study period (n = 676), 191 (27.4%) showed signs of envenoming. Sixty-two presented with signs of local envenoming only (11 in Damak and 51 in Charali), 47 presented with signs of systemic envenoming only (30 in Damak and 17 in Charali), and 82 presented with signs of both local and systemic envenoming (63 in Damak and 19 in Charali).
- Patients bitten by a krait had significantly higher chances to be put under mechanical ventilation (OR = 14.933, 95% CI: 1.693-132.52, p=0.01) and transferred to an intensive care unit (OR = 5.813, 95% CI: 1.051-32.145, p=0.049) compared to those bitten by a cobra.
