# Supplementary material for: Use of Molecular Diagnostic Tools for the Identification of Species Responsible for Snakebite in Nepal: A Pilot Study
Source: PLoS Negl Trop Dis. 2016 Apr 22;10(4):e0004620. doi: 10.1371/journal.pntd.0004620 (PMC4841570; doi:10.1371/journal.pntd.0004620)
Supplement: S2 Text — (PDF) [file pntd.0004620.s003.pdf]

Standard Operating Procedure for collecting swab samples		Hôpitaux Universitaires de Genève	
SOP Snake N° 04		**Name**	**Date and signature**
Version N° 2	**Author**	E. Alirol	01/03/2012
Version date: 01 March 2012	**Reviewed by**	S. Sharma/ F. Chappuis / U. Kuch	01/03/2012
Previous versions: 24 May 2011	**Approved by**	B. Taylor	01/03/2012

Purpose

When a snake bite victim is admitted to the health centre, it is of primary importance to determine which snake is responsible for the bite. In fact the appropriateness and efficacy of antivenom depends on the species. Unfortunately prospective species determination is not possible because no bed-side diagnostic test exists yet. In the present study, one swab sample and one blood sample will be collected from each study participant. This will enable us 1) to establish how many snake species cause neurotoxic envenoming in the 3 study centres and 2) to determine retrospectively if the identity of the snake species is correlated with antivenom treatment outcome.

The purpose of this SOP is to describe how to collect, process and store swab samples from study participants. It also gives instructions on how to ship samples to BPKIHS.

Responsibilities

Person	Activities
Site Investigator	- Collect swab samples from snake bite victims<br>- Store swab samples in appropriate place<br>- Keep a record of all samples collected<br>- Ship samples to BP Koirala Institute of Health Science (BPKIHS)
Monitor	- Verify that samples are appropriately stored and labelled.<br>- Verify that samples records are up-to-date and accurate

Detailed Procedure

1- Swab sample collection

Swabbing of skin at the bite site should be performed as soon as possible after admission and after presence of neurotoxicity is confirmed.

Identify bite site on victim's body. Physically remove any objects that cover the bite site (e.g., ligature, plant leaves, dressing, soil, etc.)

Look for puncture or scratch marks made by the snake's teeth.

Do not wash or disinfect bite site before swab samples are taken. If the patient does not show signs of neurotoxicity upon admission, clean the bite site and do not wait for neurotoxicity to appear.

Use one swab-stick with cotton swab per patient. Swab-sticks must be new and clean.

First put a drop of distilled water on the swab.

Then perform swabbing by intense, multiple rubbing of all sides of the cotton swab:

→ on all visible puncture or scratch marks of the bite,

→ and an area of about 1 cm around any such mark

Place swabs back in their collection tube and screw caps.

Label tube with patient n°, date and time, and study centre name. Use pen. Do not write the name of the patient on the tube.

Write the swab n°, date and time of collection in the Case Report Form (CRF) on page 6.

2- Sample storage

The swabs can be stored at room temperature. Store the swab samples on a rack/ in a box, in a locked cupboard. When the box is full, close it with tape and label it with the centre's name and a unique number (1,2,3,...).

Make sure that access to the samples is restricted to study staff only.

3- Sample Records

Record the patient n°, the date of collection and the Box n° in which the swab is stored in the "Record of Swab Samples Collected" form.

4- Shipment of samples to BPKIHS

At least once a week, and whenever a car travels from the study centre to BPKIHS, send the samples to the Principal Investigator.

Swab samples can be sent at room temperature.

Make sure the packaging of samples is such that damage does not occur during transport. Ensure the samples do not move or open during transport.

Write the date of shipment down in the "Record of Samples Collected" forms.

The day after, call the Principal Investigator to make sure he has received the samples and that they are not damaged. Write the date of receipt on the "Record of Samples Collected" forms.
