# Supplementary material for: Use of Molecular Diagnostic Tools for the Identification of Species Responsible for Snakebite in Nepal: A Pilot Study
Source: PLoS Negl Trop Dis. 2016 Apr 22;10(4):e0004620. doi: 10.1371/journal.pntd.0004620 (PMC4841570; doi:10.1371/journal.pntd.0004620)
Supplement: S1 Table — (DOCX) [file pntd.0004620.s004.docx]

**Table S1: Comparison of baseline characteristics, circumstances of the bite and first-aid measures between snake bite victims with identified (n=194) and unidentified (n=555) snake species**

| **Patient characteristics** | **Species identified (n=194)** | **Species not identified (n=555)** |
| --- | --- | --- |
| Sex |  |  |
| Female | 92 (47.4%) | 264 (47.6%) |
| Male | 102 (52.6%) | 291 (52.4%) |
| Age |  |  |
| Median in years (IQR) | 30.80 (18-40) | 30 (10-45) |
| Children (≤15 years) | 28 (14.4%) | 68 (12.3%) |
| Adults | 166 (85.6%) | 487 (87.7%) |
| Occupation |  |  |
| Farmer | 75 (39.9%) | 263 (48.8%) |
| Student | 58 (30.9%) | 146 (27.1%) |
| Housewife | 37 (19.7%) | 87 (16.1%) |
| Commercial | 3 (1.6%) | 12 (2.2%) |
| Driver | 4 (2.1%) | 8 (1.5%) |
| None | 0 | 1 (0.2%) |
| Other | 11 (5.9%) | 22 (4.1%) |
| Season of bite |  |  |
| Dry season (October to May) | 83 (42.8%) | 182 (32.8%) |
| Rainy season (June to September) | 111 (57.2%) | 373 (67.2%) |
| Time of bite |  |  |
| Night (18:00 to 05:59) | 111 (57.5%) | 276 (51%) |
| Day (06:00 to 17:59) | 82 (42.5%) | 265 (49%) |
| Transport used to reach centre |  |  |
| Motorcycle | 90 (63.8%) | 275 (60.7%) |
| Ambulance | 32 (22.7%) | 92 (20.3%) |
| Public transport | 13 (9.2%) | 48 (10.6%) |
| Car | 2 (1.4%) | 31 (6.8%) |
| Other | 4 (2.8%) | 7 (1.5%) |
| Location at time of bite |  |  |
| Indoors | 64 (33%) | 134 (24.2%) |
| Outdoors | 130 (67%) | 420 (75.8%) |
| Activity at time of bite |  |  |
| Walking outdoors | 76 (39.4%) | 150 (27.1%) |
| Working in the field | 26 (13.5%) | 113 (20.4%) |
| Working elsewhere | 28 (14.5%) | 83 (15%) |
| Resting indoors | 29 (15%) | 80 (14.5%) |
| Collecting grass/wood | 14 (7.3%) | 84 (15.2%) |
| Playing | 4 (2.1%) | 10 (1.8%) |
| Feeding cattle | 4 (2.1%) | 9 (1.6%) |
| Bathing or fishing | 3 (1.6%) | 6 (1.1%) |
| Other | 9 (4.7%) | 18 (3.3%) |
| Visited traditional healer |  |  |
| Yes | 4 (2.1%) | 47 (8.5%) |
| No | 190 (97.9%) | 508 (91.5%) |
| Applied first aid measures |  |  |
| Yes | 174 (89.7%) | 503 (90.8%) |
| No | 20 (10.3%) | 51 (9.2%) |
| Type of first aid measure |  |  |
| Tourniquet | 170 (87.6%) | 494 (89.2%) |
| Ingested chilly | 6 (46.2%) | 18 (31.6%) |
| Applied herbs to bite site | 1 (7.7%) | 19 (33.3%) |
| Bandage | 4 (30.8%) | 10 (17.5%) |
| Incisions | 0 | 7 (12.3%) |

Missing values are n=22 (occupation), n=15 (time of bite), n= 155 (transport), n=1 (location), n=3 (activity at time of bite), n=1 (applied first aid)

IQR: Interquartile range
