# Supplementary material for: Use of Molecular Diagnostic Tools for the Identification of Species Responsible for Snakebite in Nepal: A Pilot Study
Source: PLoS Negl Trop Dis. 2016 Apr 22;10(4):e0004620. doi: 10.1371/journal.pntd.0004620 (PMC4841570; doi:10.1371/journal.pntd.0004620)
Supplement: S2 Table — (DOCX) [file pntd.0004620.s005.docx]

**Table S2: Comparison of clinical features on admission of victims of snake bite with identified (n=194) and unidentified (n=555) snake species**

| **Patient characteristics** | **Species identified (n=194)** | **Species not identified (n=555)** |
| --- | --- | --- |
| Envenoming |  |  |
| Local signs | 4 (5.6%) | 58 (30.1%) |
| Systemic signs | 38 (53.5%) | 75 (38.9%) |
| Both | 29 (40.8%) | 60 (31.1%) |
| None | 123 (63.4%) | 362 (65.2%) |
| Level of consciousness |  |  |
| Alert | 191 (98.5%) | 543 (97.8%) |
| Responsive to voice | 2 (1%) | 10 (1.8%) |
| Responsive to pain | 1 (0.5%) | 1 (0.2%) |
| Unresponsive | 0 | 1 (0.2%) |
| Symptoms on admission (as reported by the patient) |  |  |
| Vomiting | 21 (10.8%) | 63 (11.4%) |
| Diarrhea | 4 (2.1%) | 5 (0.9%) |
| Difficulty in breathing | 4 (2.1%) | 14 (2.5%) |
| Pain | 76 (39.2%) | 246 (44.4%) |
| Double vision | 2 (1%) | 12 (2.2%) |
| Local signs on admission |  |  |
| Fang marks | 190 (97.9%) | 532 (95.9%) |
| Swelling | 43 (22.2%) | 171 (30.8%) |
| Local bleeding | 60 (30.9%) | 106 (19.1%) |
| Ecchymosis | 18 (9.3%) | 28 (5.1%) |
| Necrosis | 8 (4.1%) | 21 (3.8%) |
| Bullae | 1 (0.5%) | 9 (1.6%) |
| Palpable regional lymph node | 3 (2.1%) | 15 (3.3%) |
| Haemotoxic signs |  |  |
| Incoagulable blood | 7 (3.6%) | 16 (2.9%) |
| Bleeding from intravenous puncture site | 1 (0.5%) | 0 |
| Neurotoxic signs and symptoms |  |  |
| Inability to frown | 9 (4.6%) | 31 (5.6%) |
| Bilateral ptosis | 54 (27.8%) | 103 (18.6%) |
| Inability to open mouth | 3 (1.5%) | 19 (3.4%) |
| Inability to protrude tongue | 5 (2.6%) | 22 (4%) |
| Inability to swallow | 26 (13.4%) | 63 (11.4%) |
| Muscle weakness | 0 | 5 (0.9%) |
| External ophthalmoplegia | 0 | 7 (1.3%) |
| Pupil not reacting to light | 12 (6.2%) | 31 (5.6%) |
| Speech difficulties | 18 (9.3%) | 60 (10.8%) |
| Broken neck sign | 0 | 7 (1.3%) |
| Gag reflex loss | 0 | 2 (0.4%) |

Missing values are n=1 (pain), n=1 (difficulty in breathing), n=1 (incoagulable blood), n=1 (pupil not reacting to light), n=2 (external ophthalmoplegia), n=2 (double vision), n=2 (ecchymosis), n=154 (palpable regional lymph nodes)
